# Aerosol components associated with hospital mortality in systemic sclerosis: an analysis from a nationwide Thailand healthcare database

**DOI:** 10.1038/s41598-021-87114-0

**Published:** 2021-04-12

**Authors:** Chingching Foocharoen, Udomlack Peansukwech, Patnarin Pongkulkiat, Ajanee Mahakkanukrauh, Siraphop Suwannaroj

**Affiliations:** 1 Department of Medicine, Faculty of Medicine, Khon Kaen University, Khon Kaen, 40002 Thailand; 2 Chronic Kidney Disease Prevention in the Northeast of Thailand (CKDNET), Khon Kaen University, Khon Kaen, 40002 Thailand

**Keywords:** Environmental sciences, Rheumatology, Risk factors

## Abstract

Occupational and environmental associations with systemic sclerosis (SSc) have been confirmed; however, the association between aerosol components and mortality is uncertain. The study aimed to define the association between aerosol components and hospital mortality among Thai SSc patients. A study was conducted using a national database of patients covered by the National Health Security Office, hospitalised between 2014 and 2018. Data included all patients over 18 having a primary diagnosis of SSc (ICD-10: M34). Spatial resources used map information based on GPS coordinates of Thailand. Aerosol components—including organic carbon, black carbon, dust particulate matter diameter < 2.5 µm (PM2.5), and sulfate—were assessed using the NASA satellite MERRA-2 Model M2TMNXFLX v5.12.4. Spatial modelling with R Package Integrated Nested Laplace Approximation (R-INLA) was used to analyse the association between the incidence of mortality and the 5-year accumulation of each aerosol component adjusted by age, sex, and comorbid diseases. The study included 2,094 SSc patients with 3,684 admissions. Most (63.8%) were female. During admission, 1,276 cases died. R-INLA analysis indicated an increase of 1 µg/m^3^ of dust PM2.5 was associated with a respective increase in the risk of overall mortality and death due to pneumonia of 96% and 79%. An increase of 1 µg/m^3^ of dust PM2.5 resulted in 1.17, 1.18, 1.64, and 2.15 times greater risk of mortality due to pulmonary fibrosis, cardiac involvement, renal involvement, and cancer, respectively. Aerosol components—particularly dust PM2.5 exposures—increased the risk of overall, cardio-pulmonary-renal, and cancer mortality among SSc patients.

## Introduction

Systemic sclerosis (SSc) is a rare disease for which the hallmark symptom is thickened skin. Thickened skin is the classic presentation during the indurative phase of the disease. The extent of skin thickness is classified into two major subsets—limited cutaneous systemic sclerosis (lcSSc) and diffuse cutaneous systemic sclerosis (dcSSc)^1^. The skin thickness in lcSSc is limited to the face, hands, feet, forearms, and legs, while in dcSSc, the skin thickening extends to the trunk and both extremities. Rapid skin thickness progression is reported in dcSSc patients^2^, and this is associated with poor survival outcomes and development of a scleroderma renal crisis within the first two years of the disease^2^.

The cause and pathogenesis of the disease are not well understood. Genetics is a predisposing factor, and environment is thought to be associated with disease development. Possible environmental agents associated with SSc are silica dust, solvent, epoxy resins, pesticides, aromatic hydrocarbons, aliphatic chlorinated and non-chlorinated hydrocarbons, and drugs (e.g., bleomycin)^3^.

Aerosol components have been studied and are thought to be an associated factor of disease development^4^. SSc has been analysed in systemic autoimmune rheumatic diseases, but there has been no focused study on SSc vis-à-vis aerosol components. PM2.5 exposure in Quebec and Alberta, Canada, was associated with systemic autoimmune rheumatic diseases^4^. Another study—using a questionnaire on work and environmental exposure and obtaining benzene/PM10 concentration levels from the Regional Environmental Protection Agency monitors—confirmed the association between SSc and exposure to benzene or particulate matter ≤ 10 µm in diameter (PM10)^5^. The study reported on the association between benzene and scleroderma development: the concentration level was significantly correlated with diffuse skin thickness and low DLCO, but the association was not found for PM10^5^.

The association between aerosol components and SSc in previous studies^4,5^ was not strong. It is possibly related to the methods of the study, including a) the inability to determine the exact concentration of the aerosol components, which can wax and wane and be subject to the weather; b) the exposure to aerosol components was from memory, which can be faulty; and, c) the duration of exposure was difficult to determine. To date, there have been only a few studies on the cause and effect of exposure to aerosol components among SSc patients, and their assessment is challenging because of the variability of aerosol components exposure among workers and/or career changes. Our study was designed to evaluate the association between aerosol components and SSc in a specific condition (hospitalisation) and specific outcome (mortality). If the environmental factors are associated with hospital mortality in SSc patients and the distribution of the disease related to these factors is clarified, environmental management could reduce hospital costs and mortality.

## Methods

We investigated the spatial and temporal associations for hospital mortality and the relative risk of aerosol component exposure in Thailand between 2014–2018.

### Data collection

- Patient data were extracted from a national database of hospitalised patients covered by the National Health Security Office (NHSO) between 2014 and 2018. The respective number of hospitalisations in 2014, 2015, 2016, 2017, and 2018 was 5.74, 5.78, 6.06, 6.03, and 6.22 million^6^. The inclusion criteria were all patients over 15 having a primary diagnosis of SSc (ICD-10: M34) and being admitted between January 1, 2014, and December 31, 2018. The hospitalised SSc patients' demographic data, hospital addresses (province), principle diagnoses, and causes of death were evaluated.
- Spatial resources were based on the Geographical Information System (GIS). Longitudes were between 97.96852 and 105.22908. Latitudes were between 5.77434 and 20.43353^7^. The data for all of the 77 provinces in Thailand were included.
- Aerosol component data were assessed using The Modern-Era Retrospective analysis for Research and Application version 2 (MERRA-2). MERRA-2 is a NASA atmospheric satellite model (M2TMNXFLX v5.12.4) used for establishing outcomes. Analyses of aerosol components were based on the data collection and report by NASA. Each aerosol component was illustrated and evaluated for geophysical parameters in both the vertical and horizontal grids based on Giovanni’s data product between 2014 and 2018. The monthly mean level of each aerosol component was used for our evaluation. The Aerosol Diagnostics Model was used to identify each aerosol component's chemical type, including black carbon surface mass concentration, organic carbon surface mass concentration, dust surface mass concentration, and sulfate surface mass concentration.

The distribution of hospital mortality and aerosol components are shown on a map of Thailand. Bayesian modelling was used to examine space–time differences in the population and determine the etiologic risk factors and confounders.

The causes of death were classified into 2 groups—SSc-related death and non-SSc-related death. SSc-related death was defined by the causes of death recorded by the attending physician and included unspecified organ involvement, cardiac involvement, pulmonary fibrosis, renal involvement, and pulmonary arterial hypertension. The patients defined as having died due to SSc itself without specified organ involvement were described and classified as having died due to SSc-related death with unspecified organ involvement. Non-SSc-related death included the causes of death other than those defined in the SSc-related death and included infection, natural death, cancer, and chronic kidney disease. We observed a low prevalence and clinical irrelevance of specific antibodies associated with ILD, PAH, and renal crisis among the Thai population^8,9^. We thus explored the association between aerosol components and hospital mortality among SSc patients by evaluating the clinical association based on SSc's organ involvement but omitted autoantibody into the analysis.

### Statistical analysis

All databases recording a diagnosis of SSc (following thee ICD-10) were reviewed. Clinical characteristics of patients were investigated. The categorical data are presented as numbers and percentages and the continuous data as a means with standard deviations (SD) or medians and interquartile ranges (IQR), as appropriate. Data were divided into dichotomous, polytomous variables, or continuous variable, as appropriate.

As for defining the SSc distribution, the addresses on the hospitalisation date were mapped by latitude and longitude using spatial data analyses. The incidence rate ratio (IRR) and its 95% confidence interval (95%CI) were calculated to determine hospital mortality incidence in SSc. The zero-inflated Poisson regression model was used for defining the association between the incidence of mortality and the 5-year accumulation of each aerosol component adjusted by age, sex, and comorbid diseases. According to data from http://www.healthdata.org/thailand, the following conditions were the most common causes of death and/or risk factors among the Thai population, we therefore adjusted those regarding confounders (comorbid diseases) as coded by the ICD10: diabetes mellitus (E11.0, E13.0, E14.0, N08.3), hyperlipidaemia (E78.0-E78.5), hypertension (I10-I15), ischemic heart disease (I20-I25), cerebrovascular disease (I60-I69), malignant neoplasm (C00-C97), and chronic kidney disease (N18). Spatial modelling with R Package Integrated Nested Laplace Approximation (R-INLA) was applied to assess the final model of the association between the incidence of mortality and the 5-year accumulation of each aerosol component adjusted by age, sex, and comorbid diseases.

The INLA method was used to determine the spatial fields and confirm the aerosol components that were risk factors most highly correlated with hospital mortality. Spatial-Bayesian inference with Poisson log-linear modelling was used to evaluate the correlations between mortality IRR and spatial designs for individual risk. All data analyses were performed using program R version 3.6.1 (St. Louis, Missouri, USA). A *P*-value of < 0.05 was considered statistically significant.

### Ethics approval

The Human Research Ethics Committee of Khon Kaen University reviewed and approved the study as per the Helsinki Declaration and the Good Clinical Practice Guidelines (HE621357). The need for informed consent was waived by The Human Research Ethics Committee of Khon Kaen University. Our data were obtained from the 2 public domains open for noncommercial purposes. All data were anonymised for confidentiality.

## Results

All 2,094 SSc patients and their respective 3,684 admissions between 2014 and 2018 were included in the study. Most of the patients (1,336 cases, 63.8**%)** were female. The median age was 58 years (IQR 50–66). During admission, 1,276 cases died, of whom 797 (62.5%) were female. SSc-related death occurred in 758 cases (59.4%) cases. The most common causes of death among SSc-related death was SSc unspecified organ involvement (379 cases; 29.7%), followed by cardiac involvement (146 cases; 11.4%), and pulmonary fibrosis (133 cases; 10.4%). The causes of death among hospitalised SSc are presented in Table 1.

**Table 1:** Causes of death.

Causes of death	N = 1276 (%)
SSc-related death	758 (59.4)
Unspecified organ involvement	379 (29.7)
Cardiac involvement	146 (11.4)
Pulmonary fibrosis	133 (10.4)
Renal involvement	97 (7.6)
Pulmonary arterial hypertension	3 (0.2)
Non-SSc-related death	518 (40.6)
Pneumonia	171 (13.4)
Septicemia	109 (8.5)
Natural death	66 (5.2)
Cancer	39 (3.1)
Coronary artery disease	24 (1.9)
Chronic kidney disease	15 (1.2)
Gastrointestinal bleeding	9 (0.7)
Unknown causes	3 (0.2)
Others	82 (6.43)

*SSc* systemic sclerosis.

**Figure 1:**
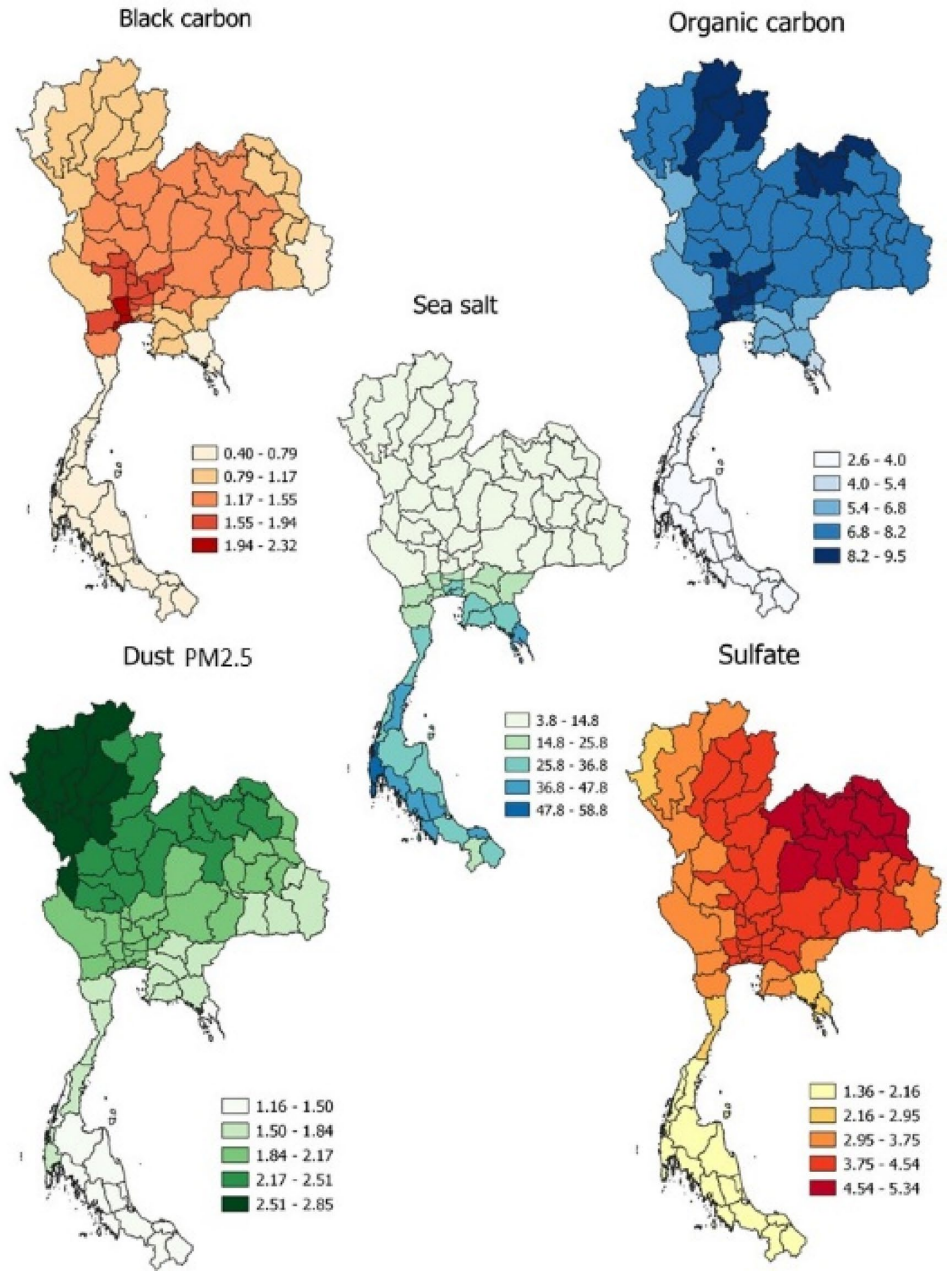
Distribution of aerosol components (QGIS software version 3.8.2: an open source and free software of geographic data system information was used for generating the maps; https://www.qgis.org/en/site/).

**Figure 2:**
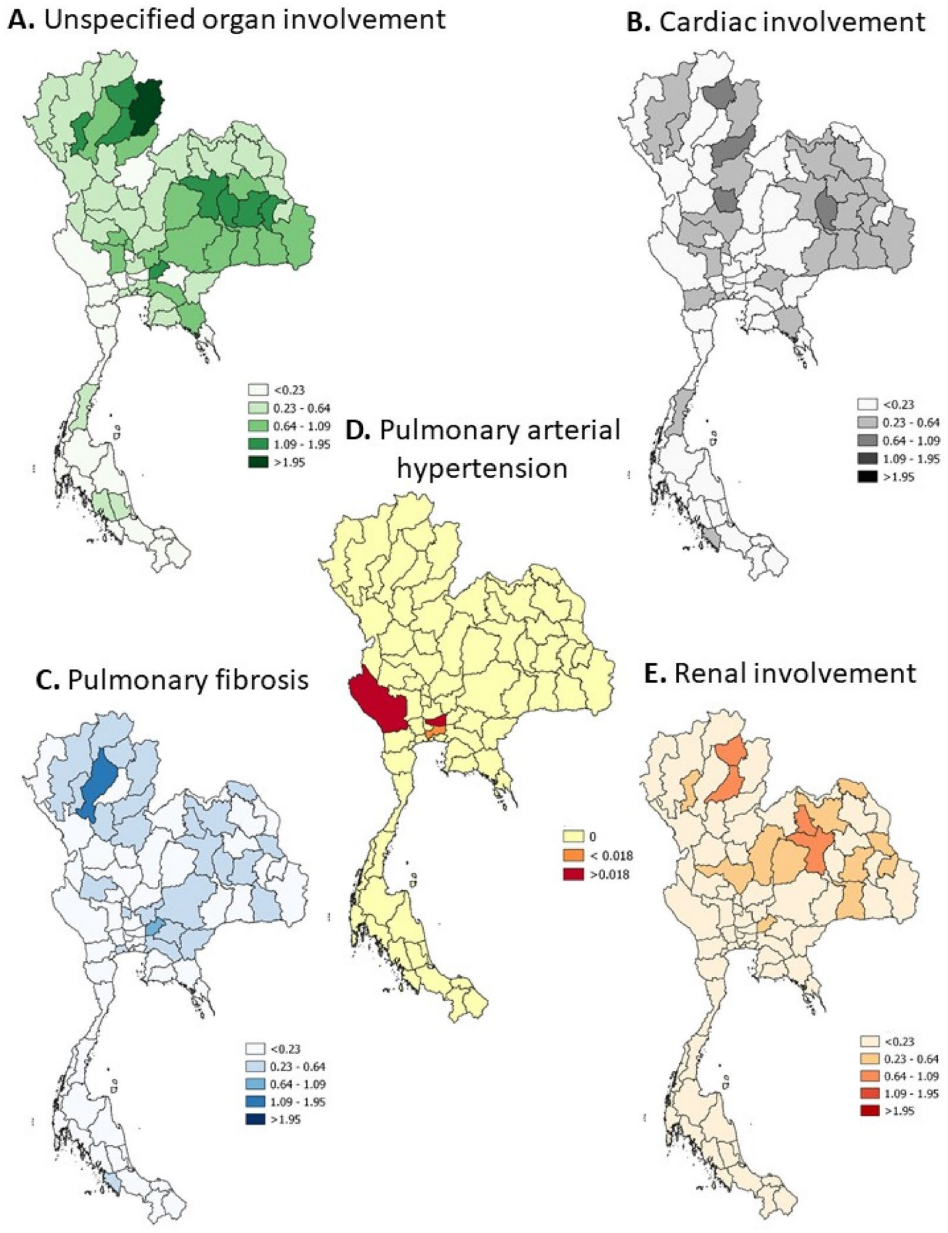
Incidence of SSc-related death stratified by province (per 100,000 dead cases) (QGIS software version 3.8.2: an open source and free software of geographic data system information was used for generating the maps; https://www.qgis.org/en/site/).

**Figure 3:**
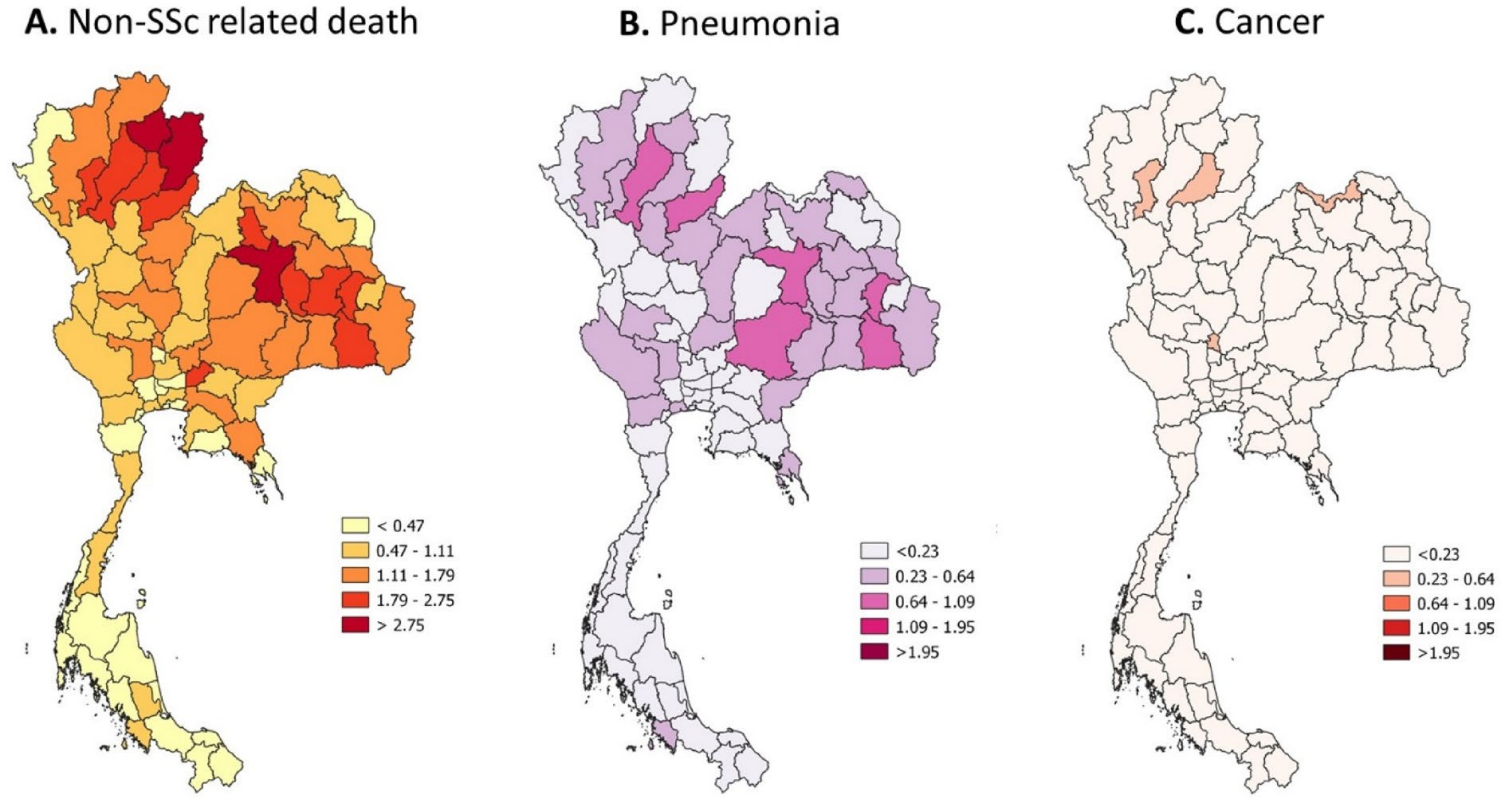
Incidence of non-SSc-related death stratified by province (per 100,000 dead cases) (QGIS software version 3.8.2: an open source and free software of geographic data system information was used for generating the maps; https://www.qgis.org/en/site/).

The baseline accumulated aerosol components from 2014–2018—which affected the hospitalisation of SSc patients and mortality among hospitalised SSc patients—are presented in Table 2. The distributions of aerosol components in Thailand are presented in Fig. 1. The respective incidence of SSc-related death and non-SSc-related death (per 100,000 dead cases) stratified by province are presented in Figs. 2 and 3.

**Table 2:** Baseline accumulated aerosol component between 2014 and 2018.

Aerosol component	Mean ± SD (µg/m^3^)	Median (IQR) (µg/m^3^)	Range (µg/m^3^)
Black carbon	1.15 ± 0.44	1.17 (0.78–1.42)	0.32–2.53
Organic carbon	6.76 ± 2.24	7.15 (5.45–8.37)	1.65–14.68
Dust PM2.5	2.09 ± 0.51	2.11 (1.80–2.36)	0.71–4.10
Sulfate	3.63 ± 1.20	3.89 (3.03–4.50)	1.31–6.08

*SD* standard deviation; *IQR* interquartile range; *PM2.5* particulate matter diameter < 2.5 µm.

Table 3 presents a comparison of the cumulative level of each aerosol component between 2014 and 2018; after adjusting for age, sex, and comorbid diseases-stratified by causes of death. The respective IRR of organic carbon, dust PM2.5, and sulfate was significantly associated with overall mortality among hospitalised SSc patients (viz., 1.18 (95%CI 1.11–1.25), 2.40 (95%CI 1.97–2.93) and 1.16 (95%CI 1.06–1.26)). Organic carbon, dust PM2.5, and sulfate were associated with overall mortality and SSc-related death in terms of unspecified organ involvement and renal involvement. Dust PM2.5 was the only aerosol components associated with SSc-related death in terms of pulmonary fibrosis and cardiac involvement and non-SSc-related death in terms of pneumonia, while organic carbon and dust PM2.5 were associated with mortality due to cancer. None of these factors was associated with mortality due to pulmonary arterial hypertension (PAH), septicemia, coronary artery disease, or chronic kidney disease (Table 3).

**Table 3:** Comparison of each cumulative aerosol component between 2014 and 2018 after adjusting for age, sex, and comorbid diseases-stratified by common causes of death.

Cause of death	IRR	P-value
**Overall mortality**		
Black carbon	1.04 (0.86–1.25)	0.71
Organic carbon	1.18 (1.11–1.25)	< 0.001*
Dust PM2.5	2.40 (1.97–2.93)	< 0.001*
Sulfate	1.16 (1.06–1.26)	0.001*
**SSc-related death**		
***Unspecified organ involvement***		
Black carbon	1.00 (0.72–1.41)	0.98
Organic carbon	1.26 (1.13–1.40)	< 0.001*
Dust PM2.5	2.33 (1.62–3.34)	< 0.001*
Sulfate	1.23 (1.05–1.44)	0.01*
***Pulmonary fibrosis***		
Black carbon	1.17 (0.66–2.06)	0.59
Organic carbon	1.16 (0.98–1.37)	0.08
Dust PM2.5	2.48 (1.36–4.51)	0.003*
Sulfate	1.20 (0.93–1.57)	0.17
***Cardiac involvement***		
Black carbon	0.97 (0.56–1.67)	0.91
Organic carbon	1.15 (0.98–1.34)	0.08
Dust PM2.5	2.41 (1.36–4.27)	0.003*
Sulfate	1.16 (0.92–1.48)	0.22
***Pulmonary arterial hypertension***		
Black	1.00 (0.35–2.84)	0.99
Organic	1.00 (0.80–1.24)	0.99
Dust PM2.5	1.00 (0.42–2.35)	0.99
Sulfate	1.00 (0.65–1.53)	0.99
***Renal involvement***		
Black carbon	1.41 (0.67–2.96)	0.37
Organic carbon	1.54 (1.15–2.07)	0.004*
Dust PM2.5	5.27 (2.27–12.24)	< 0.001*
Sulfate	1.75 (1.14–2.69)	0.01*
**Non-SSc-related death**		
***Pneumonia***		
Black carbon	1.17 (0.69–1.99)	0.57
Organic carbon	1.10 (0.95–1.28)	0.19
Dust PM2.5	1.88 (1.09–3.24)	0.02*
Sulfate	1.10 (0.87–1.39)	0.42
***Septicemia***		
Black carbon	0.96 (0.52–1.80)	0.91
Organic carbon	1.03 (0.88–1.21)	0.72
Dust PM2.5	1.86 (0.99–3.49)	0.06
Sulfate	0.98 (0.75–1.27)	0.88
***Cancer***		
Black carbon	1.40 (0.45–4.37)	0.56
Organic carbon	1.67 (1.02–2.74)	0.04*
Dust PM2.5	5.86 (1.57–21.87)	0.01*
Sulfate	1.81 (0.97–3.36)	0.06
***Coronary artery disease***		
Black carbon	1.14 (0.26–4.97)	0.86
Organic carbon	1.05 (0.71–1.58)	0.80
Dust PM2.5	4.96 (0.79–31.02)	0.09
Sulfate	0.88 (0.47–1.64)	0.69
***Chronic kidney disease***		
Black carbon	0.81 (0.08–8.15)	0.86
Organic carbon	2.08 (0.73–5.97)	0.17
Dust PM2.5	5.66 (0.41–78.48)	0.20
Sulfate	1.35 (0.43–4.26)	0.61

After the fixed effect estimated by R-INLA analysis was adjusted by age, sex, and comorbid diseases, an increase of 1 µg/m^3^ in dust PM2.5 was associated with an increase of around 25% and 4% in the risk of unspecified organ involvement SSc-related death and mortality due to pulmonary fibrosis. An increase of 1 µg/m^3^ of dust PM2.5 was associated with a 96% increase in overall mortality and 79% in mortality due to pneumonia and resulted in 1.17, 1.18, 1.64, and 2.15 times greater risk of mortality due to pulmonary fibrosis, cardiac involvement, renal involvement, and cancer, respectively (Table 4).

**Table 4:** Posterior mean, posterior standard deviation, and posterior 95% credibility interval for the fixed effects of the regression model of hospital mortality risk in Thai SSc patients by aerosol components.

Type	Mean	SD	2.5%	50%	97.5%
**Overall mortality**					
Intercept	−13.480	0.268	−14.014	−13.477	−12.961
Organic carbon	0.004	0.060	−0.114	0.004	0.120
Dust PM2.5*	0.968	0.151	0.672	0.968	1.264
Sulfate	−0.035	0.074	−0.180	−0.034	0.109
**SSc-related death**					
***Unspecified organ involvement***					
Intercept	−14.378	0.509	−15.405	−14.368	−13.408
Organic carbon	0.203	0.103	−0.001	0.204	0.403
Dust PM2.5	0.443	0.273	−0.094	0.443	0.978
Sulfate	−0.122	0.134	−0.387	−0.121	0.140
***Pulmonary fibrosis***					
Intercept	−15.879	0.794	−17.499	−15.858	−14.381
Organic carbon	−0.052	0.123	−0.292	−0.052	0.191
Dust PM2.5*	1.171	0.430	0.318	1.174	2.007
***Cardiac involvement***					
Intercept	−15.722	0.743	−17.238	−15.702	−14.321
Organic carbon	−0.071	0.117	−0.301	−0.072	0.159
Dust PM2.5*	1.178	0.417	0.351	1.180	1.987
***Renal involvement***					
Intercept	−19.272	1.615	−22.730	−19.168	−16.386
Organic carbon	0.012	0.246	−0.482	0.015	0.486
Dust PM2.5*	1.638	0.604	0.468	1.634	2.836
Sulfate	0.428	0.284	−0.134	0.429	0.983
**Non-SSc-related death**					
***Pneumonia***					
Intercept	−15.603	0.703	−17.009	−15.593	−14.250
Dust PM2.5*	0.790	0.283	0.239	0.789	1.347
***Cancer***					
Intercept	−23.180	3.232	−30.274	−22.897	−17.609
Organic carbon	0.086	0.394	−0.700	0.090	0.846
Dust PM2.5*	2.154	0.962	0.335	2.130	4.115
Sulfate	0.721	0.434	−0.135	0.722	1.570

*Statistically significant.

## Discussion

This geographical study confirmed a significant association between dust PM2.5 and the mortality related to disease among hospitalised SSc patients, mainly due to pulmonary fibrosis after adjusting for age, sex, and comorbid diseases. Dust PM2.5 was also a risk of overall mortality, cardiac mortality, renal mortality, death due to pneumonia and cancer among SSc patients. According to the map, the concentration of dust PM2.5 and dust PM2.5 distribution paralleled the proportion of death cases caused by pulmonary fibrosis, cardiac involvement, renal involvement, and pneumonia.

Aerosol components are a complex mixture of particulates from vehicles, biomass or agricultural combustion, industry, generation of power, and other natural sources. Burning biomass, long-distance transportation, traffic, and secondary aerosol production are the principal sources of organic carbon production^10^. In the context of air pollution, organic carbon and black carbon are principle components of PM—particularly PM2.5 and PM10, widespread in the air. The components of PM include both chemical and biological components. The typical chemical components are organic carbon, sulfates, nitrates, ammonium, metals particle-bound water, polycyclic aromatic hydrocarbons, and inorganic ions. The common biological components are allergens and microbial compounds^11^. It is difficult to evaluate the aerosol components' effect on health separately because of co-linearity and the mixture of aerosol components during exposure. Consequently, the interpretation of any association between the effect on health and each aerosol component should be made with caution.

The health effects of inhaled PM on respiratory and cardiovascular morbidity and mortality have been well documented^12,13^. Inhaled PM can aggravate respiratory symptoms, particularly asthma, increase hospitalisation, and increase mortality from respiratory disease, cardiovascular disease, and lung cancer^11,12,14–16^. Our findings support the conclusion of an increased risk of cardiopulmonary mortality among SSc patients by aerosol components exposure. Although there are reports on the impact of dust exposure on cardiopulmonary mortality^12,13^, the association between dust PM2.5 and renal mortality has not been documented. We are the first report to confirm the association between dust PM2.5 exposure and renal mortality. Dust PM2.5 exposure affects cellular respiration, but the precise mechanism on renal cells is uncertain. In an animal model, renal fibrosis (glomerulosclerosis and tubulointerstitial fibrosis) was found in rats with industrial dust exposure^17^. Dust PM2.5 exposure might also promote renal fibrosis in human SSc. Due to limited data, the effect of dust PM2.5 on renal cells should be further investigated in human.

SSc with cardiopulmonary involvement might be exacerbated by particulate matter in the breathing air. Our study revealed an association between mortality due to cardiopulmonary involvement and aerosol components (dust PM2.5). Cardiopulmonary involvements—particularly pulmonary fibrosis—are a common clinical feature in both dcSSc and lcSSc. Pulmonary fibrosis is also commonly found as a clinical presentation at disease onset^18^. The patients with cardiopulmonary involvements are more likely to have more disease severity and a higher risk of hospitalisation than those who did not have cardiopulmonary involvement^19^. Cardiopulmonary involvement is the most common cause of death among Thai SSc patients irrespective of the season^18,20^. Dust exposure has also been reported as an aggravating factor associated with poor idiopathic pulmonary fibrosis outcomes^21^. Our results indicate that air pollution or aerosol components exposure—particularly dust PM2.5—among SSc patients might aggravate disease severity by inducing inflammatory cytokines, promoting oxidative DNA damage, and increasing reactive oxygen production species that result in DNA methylation and tissue damage^22–24^.

Air pollution not only causes local pulmonary effects but also causes a systemic inflammatory reaction. The link between air pollution and systemic inflammatory oxidative processes has been reviewed^25^. The data show that inhaled fine particles can translocate to the pulmonary interstitium, promoting oxidative stress and inducing an inflammatory response by releasing pro-inflammatory cytokines and pro-thrombotic factors into blood circulation^25^. The worsening of previous inflammatory disease or conditions is associated with worsening air pollution^25^, and in our SSc patients, aerosol components were associated with higher mortality due to cardiopulmonary involvement as well as pulmonary infection (pneumonia). Oxidative stress may induce both a systemic and local inflammatory response to aerosol components, which might be the mechanism for increasing the risk of cardiopulmonary complications and mortality among SSc patients.

According to our findings, black carbon, organic carbon, and sulfate had less effect on cardiopulmonary mortality among SSc patients than dust PM2.5. These findings are comparable to the risk of lung cancer reported in previous studies^12,14^. Sulfur dioxide also had no association with rheumatoid arthritis (RA)^26^. It is unclear why black carbon and sulfate had no significant effect on SSc despite their being small-sized aerosol particles that can penetrate the lung barrier and enter the blood circulation^27^. According to the mixed aerosol and chemical components in inhaled air, it is impossible to inhale each aerosol component separately. A previous study found a synergistic effect between sulfur dioxide and PM10 vis-à-vis lung cancer^28^. Inhaling a combination of aerosol substances may thus have a synergistic effect. Notwithstanding, there has been no report on such synergistic effects in autoimmune diseases, so we cannot confirm whether there is any synergistic effect of aerosol components on SSc and its outcomes.

Dust is not only associated with most SSc-related deaths but also with non-SSc-related deaths due to cancer. Previous studies revealed an association between dust and lung cancer^14,29–31^, so dust might be a carcinogen. The evidence revealed that aerosol components promote inflammation^25^, induce reactive oxygen species production, promote DNA hypermethylation^32^, alter cell cycles, and induce cell apoptosis^33^. The alteration of cell cycles by aerosol components might promote carcinogenesis; however, the mechanism of carcinogenesis due to aerosol components exposure is unclear.

Air pollution has been reported to be associated with mortality in the Thai population. The estimated non-accidental mortality is 0.4%^12^. A recent study found a negative correlation between temperature and humidity with PM10^12^. A study from Finland reported that the sources of organic carbon in Northern Europe were high in winter^10^. The findings might reflect the high rate of heat production by biomass combustion in winter exacerbated by poor ventilation^10^; thereby affecting human health to a greater extent in winter than in other seasons. We have no data on the correlation between aerosol components and hospital mortality among SSc patients stratified by season or a comparison of hospital mortality between SSc and the general population. Nevertheless, our initial information can serve as a guide for interventional planning to reduce air pollution associated with mortality in both the general population and among SSc patients who often have systemic involvements.

According to the annual WHO air quality guideline (AQG), only 9 of the 34 members states had an AQG level of PM10 less than 20 µg/m^3^ in 2010^11^. The level represents an interim target for air pollution to promote low concentrations of air pollution in a high pollution area. Although a threshold or absolute cut-off level for safe or no health risk PM has not been defined^11^, reducing aerosol components should be promoted to protect the health of SSc patients and the general population. Improvement of health and a decline in total and/or cardiopulmonary mortality might be expected to begin soon after a reduction in air pollution.

Our study had some limitations, including (a) the database did not include other healthcare providers such as the Civil Servants’ Benefit System, the Social Security Office, or self-payment, so the database did not represent the whole Thai population, although it did include around 70% of the population; (b) the clinical information from the database was limited, so we were unable to evaluate some of the confounders (i.e., occupation, economic status, and smoking); (c) the principal diagnosis in the database was coded according to the ICD-10, but it does not provide a specific code for the dcSSc and lcSSc subsets, so we cannot compare mortality between the two; (d) the cause of death was extracted according to the ICD-10 per attending physician notes, so it is not known whether the definite cause of death was confirmed by autopsy; and, (e) it is uncertain whether the patients stayed at home or residence over the past 5 years or whether they migrated. The strengths were: (a) the study was based on an extensive database of in-patient units for SSc patients, thus providing detailed epidemiological characteristics of hospitalised SSc patients in Thailand; (b) we included major organ involvement of SSc into the analysis; (c) we performed an analysis of the area of aerosol components exposure for the province of the hospital where patients were admitted, so we were able to correlate hospitalised mortality with the area of aerosol components exposure by province and healthcare service region; and, (d) the results can be generalised to other Southeast Asian countries with similar ethnicity, climate, and aerosol components exposure.

## Conclusion

Aerosol components were associated with hospital mortality among SSc patients. Organic carbon increased the risk of hospital death due to pulmonary fibrosis and unspecified organ involvement SSc-related death, while dust PM2.5 increased hospital death risk due to cardiac involvement, pulmonary fibrosis, and overall mortality. A policy for intervention to reduce aerosol components is needed to improve health outcomes.

### Ethical consideration

The study involving human participants followed the ethical standards of the institutional and/or national research committee. The Human Research Ethics Committee of Khon Kaen University approved the study as per the Helsinki Declaration and its later amendments or comparable ethical standards and the Good Clinical Practice Guidelines (HE621357).

### Consent for publication

All of the authors consent to publication and grant the Publisher exclusive license of the full copyright**.**

## Data Availability

Data and material are available as per request.
